# Aggregation of Fuzzy Nutrient-Level Evaluations Using a Parametric T-Norm: Mathematical Properties, Calibration, and a Proof-of-Concept Analysis

**DOI:** 10.3390/nu18193151

**Published:** 2026-09-24

**Authors:** Jan Wirsam, Bernd Wirsam, Claus Leitzmann

**Affiliations:** 1 Campus Treskowallee, HTW Berlin, 10318 Berlin, Germany; wirsam@htw-berlin.de; 2 Independent Senior-Researcher, Schubertstrasse 4, 35440 Linden, Germany; 3 Institute for Nutrition, University of Giessen, 35390 Giessen, Germany

**Keywords:** fuzzy logic, nutrient intake, aggregation operator, T-norm, Schweizer operator, nutritional optimization, evaluation field, diet quality

## Abstract

**Backgroumd/Objectives:** Since 1995, the intake of individual nutrients has been evaluated using fuzzy logic, with aggregation of the resulting fuzzy sets enabling the weighing of one nutrient against another, and the evaluation and optimization of overall nutrient intake. The aggregation operator used in earlier work, defined as the product of the minimum and harmonic mean operators (MIN×H), performed well in practice but had undesirable mathematical properties: it is not associative, model extensions can shift results even when all conditions are fully satisfied, and the resulting evaluation field contains discontinuities inconsistent with biological behavior. **Methods:** We therefore reviewed the family of parametrized T-norms for an alternative and identified the Schweizer (3) operator, fitting its parameter to best match the existing operator using 246 real-world dietary records from 123 participants (a sustainability study cohort of predominantly students and young adults; mean age 25.3 ± 16.4 years; 54 male, 69 female). **Results:** Strong agreement was achieved at *p* = 6 (R^2^ = 0.9949; RMSE = 0.0187), outperforming *p* = 4 (R^2^ = 0.9852) while remaining more balanced than *p* = 8 (systematic intercept offset). The new operator satisfies all required mathematical properties and produces a smooth, simply structured evaluation field that reveals synergistic and substitution effects among foodstuffs, as well as the essentiality of certain foodstuffs under specific conditions. The resulting evaluation field shows a wide, high-dimensional plateau rather than a sharp peak, interpretable as stability, error tolerance, and a high degree of diversity, offering a new, mathematically robust perspective on healthy nutrition and the interactions among foodstuffs. **Conclusions:** This work proposes and illustrates a mathematical aggregation framework; its nutritional and clinical validity remains to be established in future studies.

## 1. Introduction

Fuzzy logic, introduced by Zadeh in 1965 [1], has since been applied across a broad range of domains where imprecision and uncertainty must be handled in a principled way. In engineering and control systems, fuzzy controllers are standard tools for complex nonlinear processes [2]. In medicine and clinical decision support, fuzzy logic is used for diagnosis, treatment selection, and patient monitoring [3]. Nutrition-related clinical decision support based on fuzzy logic is also emerging [4]. More broadly, fuzzy-logic-based decision support has been proposed for economics, pattern recognition, and environmental assessment [5].

In 1995, initial steps were taken to evaluate nutrient intake using fuzzy logic [6]. Each evaluation of a nutrient is represented by a fuzzy set defined by degrees of membership that vary between 0 and 1 as the daily intake amount of a given nutrient varies. These values (pure mathematical numbers, dimensionless scalars) were translated into linguistic variables such as “potentially life-threatening” or “optimal.” Using these linguistic variables, fuzzy sets for the intake of specific nutrients were constructed under the condition that the evaluation of all other nutrients was optimal (evaluation = 1). For practical use, the linguistic variables were further translated into the colors green, yellow, and red—and the gradient between them—to provide an intuitive visual evaluation, as shown for the fuzzy set “optimal intake of potassium” in Figure 1 as an example.

Translating nutrient-level evaluations into an evaluation of actual foodstuff intake requires a form of aggregated evaluation. In earlier work, this was accomplished using the MIN×H operator [6].

This operator has since been implemented in numerous professional nutrition consulting programs. In practice, both the fuzzy sets and the implemented operator performed satisfactorily. However, several theoretical considerations suggested room for improvement in both the fuzzy sets and the aggregation method. Some of these disadvantages were discussed in [7], which also questioned whether official recommendations—intended for population-level guidance—are appropriate for evaluating individual nutrient intake. Based on the official recommendations, an interpretation incorporating a kind of probability that a nutrient requirement is met was proposed.

Accordingly, the linguistic variables were extended with statistical modifiers such as “very probably” or “possibly,” producing expressions such as “very probably well-balanced supply of nutrient a” or “possibly critical undersupply of nutrient b.” All fuzzy sets were carefully adapted to the most current DGE reference intake values [8].

Several critical disadvantages of the aggregation operator used in [6] were identified [7]:

- The operator is not associative. While not critical in practice, associativity would simplify the algorithms used to calculate the aggregated evaluation.
- Expanding the model—for example, by adding fuzzy sets for the evaluation of an additional nutrient relevant to a special diet—changes the aggregation result even when all conditions are fully satisfied (evaluation = 1).
- Applying this aggregation across all fuzzy sets while varying foodstuff amounts produces an evaluation field with discontinuities, since the minimum value can shift abruptly from the evaluation of one nutrient to that of another. This does not reflect the behavior of a biological, physiological system.

The aim of the present work was therefore to identify an aggregation operator that resolves these disadvantages while remaining closely aligned with the results of the operator already validated through extensive practical use, offering a new, mathematically robust perspective on healthy nutrition.

### Existing Approaches to Nutrient-Level Aggregation

Several methods have been proposed over the past two decades to aggregate individual nutrient evaluations into a single diet-quality score. The most widely used are the Nutrient Adequacy Ratio (NAR) and the Mean Adequacy Ratio (MAR), in which each nutrient’s intake is expressed as a fraction of its recommended value (capped at 1.0), and the resulting ratios are averaged arithmetically [9]. A more recent development is the Probability of Adequate Nutrient Intake index (PANDiet), which aggregates adequacy probabilities for 24 nutrients into a single score using two sub-scores for under- and over-consumption [10]. The Total Nutrient Index (TNI) extends this approach by including nutrient contributions from dietary supplements alongside food intake [11]. Food-based indices—such as the Healthy Eating Index, the Alternative Healthy Eating Index, and the Global Diet Quality Score—operate at the level of food groups rather than individual nutrients, making them simpler to compute but less sensitive to specific micronutrient shortfalls [12].

All these methods share a common mathematical limitation: they aggregate individual nutrient evaluations by simple averaging, which allows a critical deficiency in one nutrient to be offset by abundance in others. A diet with zero intake of an essential nutrient will not receive a zero overall score under MAR or PANDiet, because the remaining nutrients continue to contribute positively to the mean. The fuzzy logic framework applied here addresses this directly: the T-norm aggregation operator has an absorbing element of 0, meaning that a single evaluation of 0 forces the total aggregated evaluation to 0 regardless of all other nutrient scores. Beyond this property, the resulting “evaluation field” is a continuous, differentiable surface whose topology encodes mathematically defined relationships among foodstuffs—synergy, substitution, and essentiality—that are invisible to averaging-based scores and that are consistent with, though not validated against, physiological mechanisms.

Earlier fuzzy-logic-based work in nutrition has applied fuzzy sets at the food-group level [13], optimizing recommended servings within a food pyramid rather than evaluating nutrient intakes directly. The present approach operates at the nutrient level and addresses the mathematically critical question of how nutrient-level evaluations should be combined—a question that the choice of aggregation operator answers in a formally rigorous and empirically calibrated way.

A further motivation for applying fuzzy logic to nutrition is that nutrition itself is an inherently imprecise system at multiple levels. The nutrient content of individual foods varies with cultivar, growing conditions, storage time, and preparation method, and can therefore only be represented approximately in food composition databases. Dietary intake data from records or 24-h recalls are subject to recall bias, portion size estimation error, and day-to-day variability. The official nutrient reference values themselves carry epistemic uncertainty: many are Estimated Average Requirements (EAR) or Adequate Intakes (AI) derived from limited population data and are intended as population-level guidance rather than precise individual thresholds [8]. Fuzzy logic, with its capacity to represent graded degrees of membership rather than binary adequacy/inadequacy boundaries, is thus not merely a mathematical convenience but a methodologically appropriate response to the inherent imprecision of nutritional data and recommendations.

## 2. Materials and Methods

### 2.1. Selection of an Aggregation Operator from the T-Norm Family

To address the disadvantages identified above, we examined alternative approaches and reviewed operators from the T-norm family [14].

A T-norm is a generalized conjunction—specifically, a monotone (not to be confused with monotonicity in analysis), associative, and commutative [0, 1]^2^ → [0, 1] mapping with neutral element 1 and absorbing element 0. Because of associativity, a T-norm yields a mapping from an n-dimensional space to a single number. The existence of the neutral element 1 means that, in our application, full satisfaction of one nutrient’s recommendation does not alter the aggregation of all other qualities: the original fuzzy set for a given nutrient is reproduced whenever the conditions defining that fuzzy set are met. The existence of the absorbing element 0 implies that a single evaluation of 0 forces the aggregated value to 0 as well. In terms of linguistic variables, this means that if one nutrient is evaluated as “probably life-threatening,” the aggregation of all qualities carries the same verbal interpretation, regardless of how favorable the other qualities are. The result of a T-norm is always less than or equal to the minimum of its inputs.

We systematically reviewed all T-norm family operators that appeared suited to our needs [14] (e.g., minimum, algebraic product, drastic product, Hamacher product, Einstein product, and the Yager and Dubois intersection operators). None of these proved flexible enough to yield satisfactory aggregation results. We therefore turned to operators with adjustable parameters [14] (e.g., Yager, Schweizer (1), Schweizer (2), Schweizer (3), Hamacher, Frank, Dombi, Weber, and Dubois). All of these operators reduce to classical dual logic as a special case and behave like the classical operators when membership functions are restricted to the values 0 and 1. By using fuzzy sets and fuzzy operators, the model of human decision-making becomes more flexible: the binary categories of “black and white” or “true and false” are replaced by a continuum of intermediate possibilities.

Our analysis focused primarily on suitability for nutrition applications. This required proper behavior in high-dimensional decision spaces and a close relationship to the operator already validated through extensive past use. Consequently, the operator could not be the minimum operator, nor could it incorporate the minimum, given the “physiology” of the resulting evaluation field and its sensitivity to other fuzzy evaluations below 1.

After conducting these analyses, we selected the Schweizer (3) operator [15] for detailed evaluation. This operator is defined by Equation (1):

T(x,y) = 1 − [(1 − x)^p^ + (1 − y)^p^ − (1−x)^p^ × (1 − y)^p^]^1/p^  p > 0 (1)

Associativity allows this operator to be implemented as a pairwise recursive procedure: the evaluations of the first two nutrients are aggregated, and the result is then aggregated with the third evaluation, and so on; because the operator is associative, the result is independent of processing order. Writing out a full formula for this operator in a high-dimensional space is impractical, as it would contain too many terms; the computer-based calculation therefore follows a successive procedure to represent a [0, 1]^n^ → [0, 1] mapping.

### 2.2. Parameter Fitting

The task was to find a value of *p* that satisfies the following criteria:

- Close to the results of the minimum operator at high values
- Clearly below the results of the minimum operator when several fuzzy sets have low values
- Close to the results of the MIN×H operator used previously
- A resulting evaluation field topology that is consistent with the behavior of a biological system

To maximize overlap with the results of the earlier operator, we drew on a set of 246 real-world dietary records from a sustainability study [16]. The dataset comprised 123 participants (54 male, 69 female; mean age 25.3 ± 16.4 years, range 4–73; mean BMI 23.1 kg/m^2^), predominantly students and school pupils, and does not represent a general population sample. For each participant, both the original recorded diet and its numerically optimized counterpart were included, yielding 246 records in total. Mean overall evaluation of real-world diets under MIN×H was 0.364 ± 0.197 (range 0.011–0.839). The aggregation included the evaluation of 35 nutrients. The fuzzy sets used corresponded to the reference values of the DGE. For vitamin E, vitamin K, pantothenic acid, biotin, sodium, potassium, copper, and manganese, estimated values were used; for fluoride, guideline values were applied. Selenium, chromium, and molybdenum were not included in the software version used.

After optimization, the mean rose to 0.696 ± 0.140 (range 0.093–0.890). Records were processed with OptiDiet [17] using BLS 3.0 [18]. We evaluated them using both the earlier operator and the new one across a range of parameter settings for *p*. The linear regression of the Schweizer (3) operator against the MIN×H operator yielded the smallest residual variance at *p* = 6; the criteria above were correspondingly best satisfied at this value (see Figure 2).

The aggregated evaluations produced by the Schweizer (3) operator [15] at *p* = 6 closely match the results obtained with the earlier operator.

### 2.3. Dimensionality Reduction for Illustrating the Optimum

Demonstrating the properties of the aggregated evaluation directly in the full foodstuff space is impractical: a typical 7-day dietary protocol contains an average of approximately 140 distinct foodstuffs. We therefore reduced the dimensionality through an iterative procedure. Starting from a reference diet for a 40-year-old man (height 1.72 m, body weight 68 kg), each iteration began with the result of the preceding optimization step, removing any foodstuffs that had no effect on the optimization result. Iterations continued until the intake of all nutrients fulfilled the DGE recommendations (all evaluations > 0.9).

## 3. Results

### 3.1. Reduced-Dimensionality Optimum

Ultimately, only 13 foodstuffs remained in the reduced model. With fewer dimensions, the multidimensional structure of the resulting evaluation field becomes clearer, revealing certain interactions among foodstuffs.

It is striking that such a close approximation to the official recommendations can be achieved with only 13 food items. In linear algebra, a system with 140 unknowns requires at least 140 linearly independent constraints for a unique solution. In the fuzzy optimization framework used here, no such hard constraint count applies; the optimizer finds the best-scoring solution within the continuous evaluation field regardless of dimensionality. This is not the case in fuzzy logic. There also appears to be a physiological explanation: foodstuffs such as vegetables contain a wide range of nutrients in approximately the correct proportions, without adverse effects.

To determine the nutrient intake at the optimum, the optimization procedure was repeated using the OptiDiet software version 6.2 with the “standard protocol” setting. The resulting intakes of the 13 foodstuffs are listed in descending order of energy contribution in Table 1.

Table 2 presents, for each nutrient, the daily intake at the optimum, the applicable DGE reference value (recommended intake, estimated value, or guideline value), and the corresponding fuzzy evaluation score. 

To avoid misunderstanding: this example is a theoretical model of nutrition intended to illustrate interactions among foodstuffs and not a general foodstuff-based dietary recommendation. While it represents a very good supply of all nutrients, many other combinations—particularly those including a larger number of foodstuffs—can achieve similarly good or even better results.

### 3.2. Contribution of Individual Foodstuffs to the Total Result

Varying the intake of a single foodstuff while holding all others at their optimal amount reveals several distinct behaviors.

Lettuce and bread showed a particularly strong influence in our historical example: removing either foodstuff caused a marked deterioration in the aggregated result. Near the optimum, both foodstuffs maintain an extended region of favorable evaluation.

In other cases, we observed that the aggregated result drops to 0 when intake of a particular foodstuff is reduced to 0. This indicates that, within a certain low-dimensional space of foodstuffs, a single foodstuff can become essential: without it, one or more essential nutrients are missing entirely.

Fish, in the historical example, exhibits a dual role. It is nearly essential as a concentrated source of vitamin D, *n*-3 and *n*-6 fatty acids, and iodine; yet intake only slightly above the optimum level leads to excessive intake of these same nutrients.

Sausage was not part of the foodstuffs comprising the optimum. However, as shown in Figure 3, small amounts of sausage do not substantially worsen the aggregated result.

### 3.3. Evaluation Field: Synergy and Substitution

To illustrate synergistic effects between foodstuffs, we used bread and lettuce as an example. The level curves and the corresponding three-dimensional surface form a hill-shaped structure. Together, these two foodstuffs raise the evaluation of overall nutrition to 0.941, as shown in Figure 4. The maximum evaluation that either foodstuff can reach alone is considerably lower—0.742 and 0.567, respectively. This indicates that each foodstuff supplies nutrients that the other does not provide in sufficient amounts. The isolines (contour projection in Figure 4) connect points of equal aggregated evaluation, illustrating that diverse combinations of the two foodstuffs can achieve equivalent nutritional outcomes.

Among the foodstuffs forming the optimum, two—milk and yogurt—have nearly identical nutrient compositions. The level curves form straight, parallel diagonal isolines, and the 3-D plot shows an evaluation field with a ridge (Figure 5). This indicates that yogurt cannot improve the evaluation beyond what milk achieves, and vice versa: these two foodstuffs can substitute for one another without producing a synergistic effect.

## 4. Discussion

The Schweizer (3) operator with the parameter setting *p* = 6 reproduces the results of the previously used MIN×H operator with strong fidelity while resolving its principal mathematical shortcomings: it is associative, it leaves the aggregated evaluation unchanged under model extensions when all conditions are fully satisfied, and it yields a continuous evaluation field free of the abrupt discontinuities produced by a minimum-based operator.

The principal advantage of the present approach over arithmetic averaging methods such as MAR [9], PANDiet [10], or the TNI [11] lies in the mathematical properties of T-norm aggregation. Food-based indices such as the Global Diet Quality Score [12] and fuzzy-logic approaches at the food-group level [13] likewise do not address the aggregation of nutrient-level evaluations in a mathematically rigorous way. Arithmetic mean-based scores allow a severe deficiency in one nutrient to be masked by adequate intake of others. The Schweizer (3) operator, as a T-norm, has an absorbing element of 0: a single nutrient evaluation of 0 forces the total to 0, ensuring that critical deficiencies cannot be averaged away. At the same time, the parametrized structure allows a smooth and calibrated transition between the strict minimum operator and more permissive aggregations, tuned here to match the behavior of the empirically validated MIN×H operator.

Beyond these formal improvements, the operator seems to offer a substantive, model-derived account of how foodstuffs interact—one that is consistent with established nutritional knowledge but whose biological validity remains to be confirmed empirically. Optimization, in this sense, means moving from an existing real-world diet up the hill of the evaluation field—either by taking a small step in the right direction (following the gradient), which is the approach used in nutrition education, or by attempting the longer path to the top of the hill.

This top is not a sharp peak but can be a wide, high-dimensional plateau. In this case, it remains stable under small variations in the amounts of the foodstuffs needed to reach the optimum, making it error-tolerant and even allowing small amounts of less healthy foodstuffs that are not required to form the optimum. This high-dimensional plateau contains many different solutions with nearly the same optimal result—leaving considerable room for different approaches to healthy nutrition. This diversity may help to explain why different dietary patterns observed across populations and cultures can all be compatible with good nutritional status.

The synergy, substitution, and essentiality effects identified here (Section 3.2 and Section 3.3) illustrate that the aggregation operator is not merely a computational convenience but a lens through which relationships among foodstuffs become visible. These findings are consistent with established nutritional knowledge—for example, the ability of milk and yogurt to substitute for one another, or the near-essential but dose-sensitive role of fish as a source of vitamin D, *n*-3/*n*-6 fatty acids, and iodine—while providing a quantitative, model-based account of these relationships.

## 5. Limitations

The present study has several limitations that should be acknowledged.

**Dependence on reference values.** The fuzzy membership functions—and therefore the entire evaluation field—are built upon DGE reference intake values [8]. Any revision of these values directly alters the membership functions and shifts the optimum. The historical DGE 2015 potassium reference value (approx. 2000–3000 mg/day) reflected in Figure 1 was subsequently revised to 4000 mg/day in 2017; Figure 1 therefore uses the historical value intentionally.

**Calibration dataset.** The parameter *p* = 6 was calibrated on a single non-representative convenience sample (predominantly students; n = 123) without cross-validation or external validation. The calibration target was the previously used MIN×H operator, which is not itself an independently validated clinical gold standard. Generalizability to other populations, dietary cultures, or nutrient reference systems remains to be established.

**Inherent imprecision of nutritional data.** As discussed in the Introduction, nutrition is an inherently imprecise system: nutrient content varies with variety, conditions, and preparation; food composition databases introduce measurement uncertainty; and many official reference values are EAR or AI estimates. The fuzzy approach is chosen partly in response to these limitations but does not eliminate them.

**Theoretical and proof-of-concept scope.** This study presents and illustrates a mathematical aggregation framework. The 13-item reduced model is a hypothetical diet for a single theoretical individual. Model outputs are computed purely from nutrient content data and do not account for bioavailability, digestibility, food-processing-related nutrient losses, or nutrient–nutrient interactions. No biomarkers or clinical outcomes were measured.

**Data availability.** The original historical dietary records were stored only temporarily within the OptiDiet software and were not retained in retrievable digital form thereafter. All other data are available on request.

**Dietary assessment uncertainty.** All dietary records are subject to the usual limitations of self-reported dietary assessment, including recall bias, portion size estimation error, and day-to-day intake variability.

**Fuzzy membership function shapes.** The individual fuzzy membership functions were constructed using the official recommendations, expert judgment, and linguistic variables rather than being derived empirically from dose–response data. Different functional forms would yield different evaluation fields and potentially different parameter choices.

## 6. Conclusions

The Schweizer (3) operator, calibrated at a parameter setting of *p* = 6 (R^2^ = 0.9949, RMSE = 0.0187; MAE = 0.0142; 95% LoA [−0.041, +0.028]; 98% of 246 records within ±0.05 of y = x), provides a mathematically sound alternative to the previously used MIN×H operator for aggregating fuzzy nutrient-level evaluations into an overall nutrition-level evaluation. It satisfies the formal properties required of a well-behaved aggregation operator—associativity, continuity, and stability under model extension—while closely reproducing the results of the earlier operator. The topology of the resulting evaluation field shows a plateau structure that can be interpreted as stability, error tolerance, and a high degree of diversity. This study presents and illustrates a mathematical aggregation framework in a proof-of-concept setting. The parameter *p* = 6 was calibrated on a single non-representative cohort and has not been validated across other populations, nutrient reference systems, or clinical contexts. Its nutritional, clinical, and external validity remains to be established in future empirical work.

## Figures and Tables

**Figure 1:**
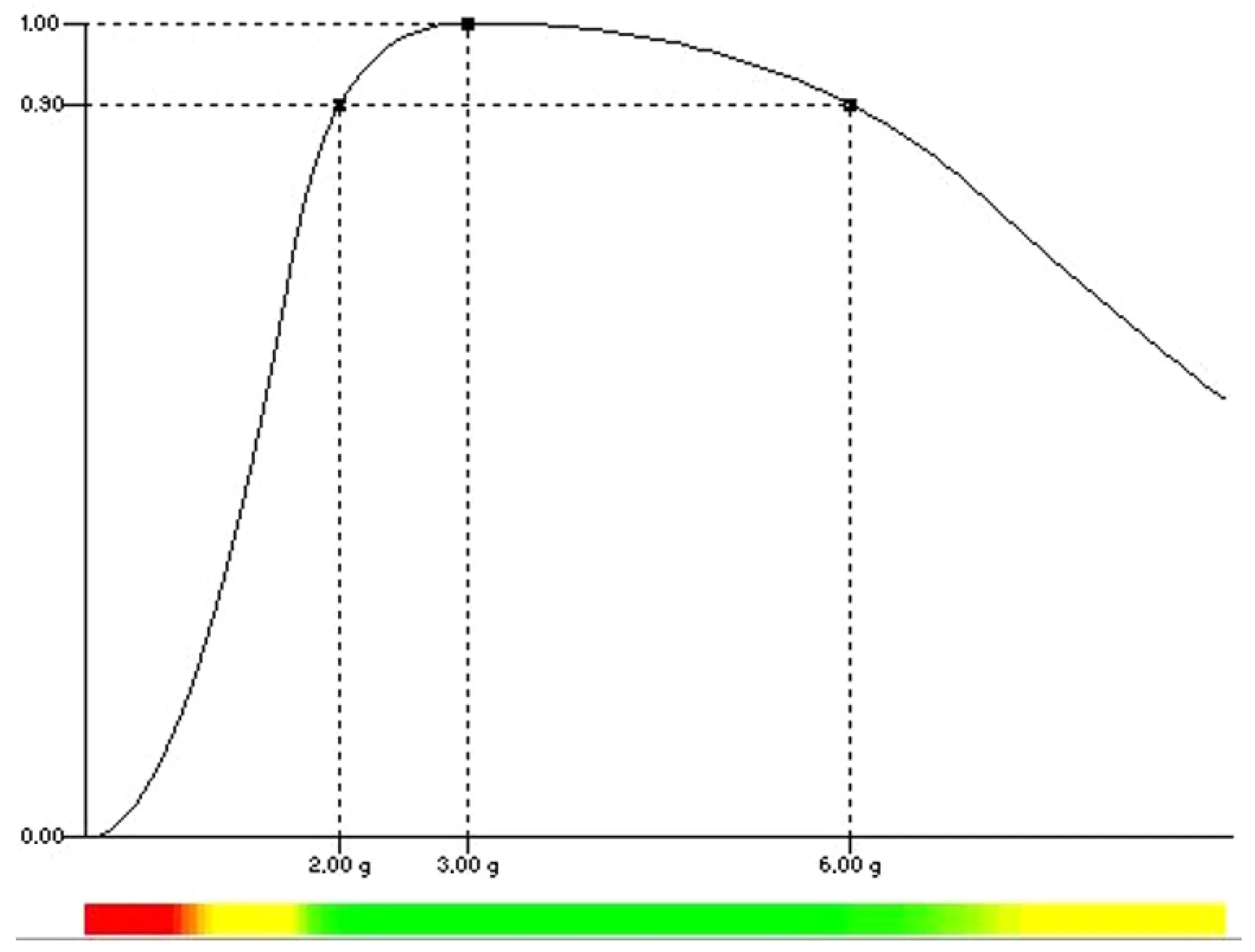
The fuzzy set for daily potassium intake, with color-coded interpretation. Note: this fuzzy set reflects the DGE potassium reference value valid at the time of the sustainability study; the reference was revised to 4000 mg/day in 2017. The colors correspond to a traffic light and provide an intuitive visual evaluation.

**Figure 2:**
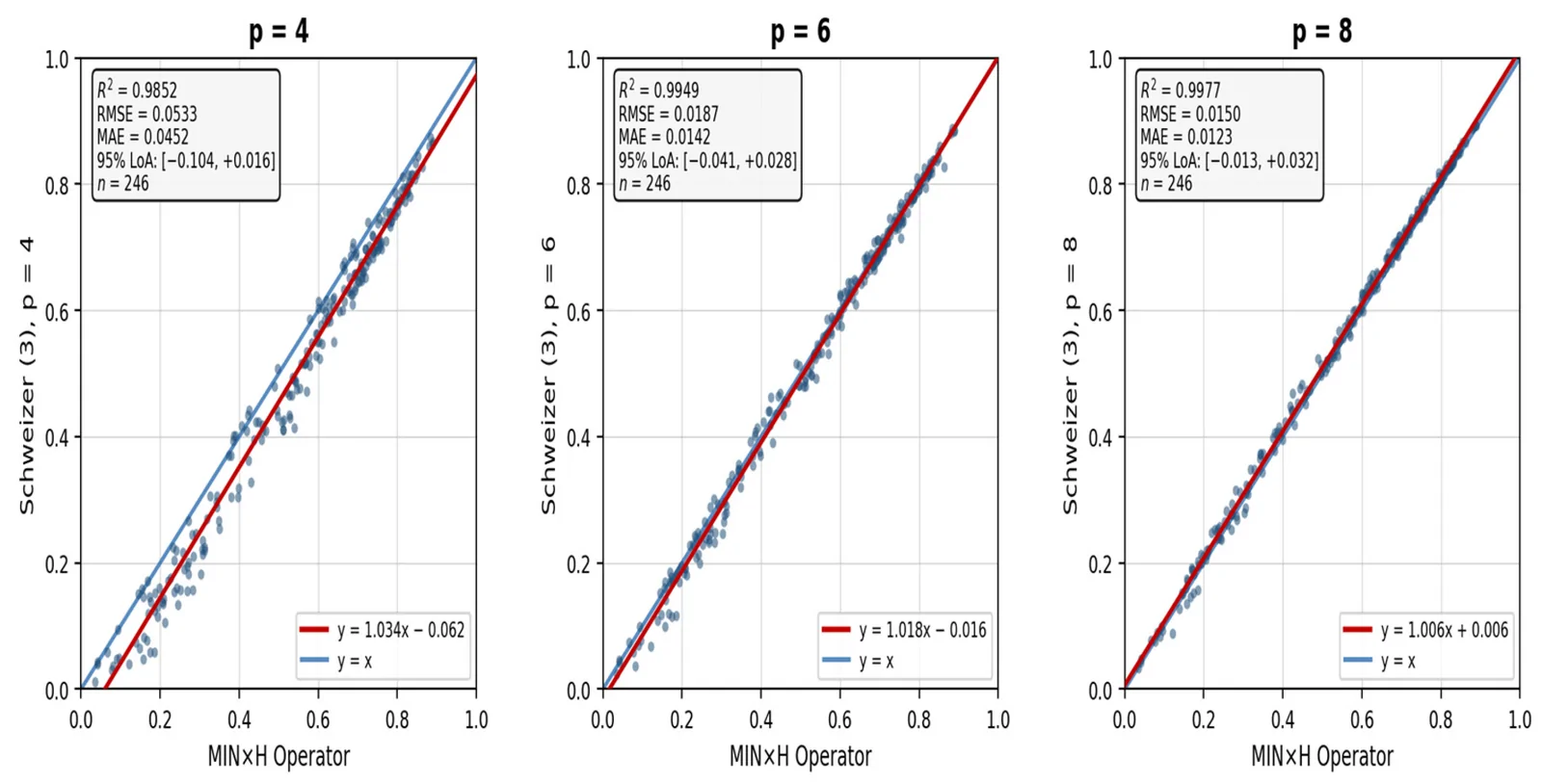
Agreement between the MIN×H operator and the Schweizer (3) operator for *p* = 4, 6, and 8, based on 246 dietary records. Red line: linear regression; blue line: identity (y = x). R^2^ and RMSE characterize the goodness of fit of the regression. Agreement with the identity line is quantified by MAE and 95% limits of agreement (LoA); for *p* = 6: MAE = 0.0142, 95% LoA [−0.041, +0.028], with 98.0% of records within ±0.05. The choice of *p* = 6 provides the best balance of R^2^, RMSE, and identity-line agreement. Eight records were excluded from the comparison because their MIN×H or Schweizer (3) output was zero, indicating that at least one nutrient had a zero fuzzy score in that record. These records were excluded after data analysis because they reflected data-entry inconsistencies in the original dietary assessment rather than genuine dietary patterns; their exclusion had no influence on the selection of *p* = 6, since the parameter fitting was robust to their inclusion.

**Figure 3:**
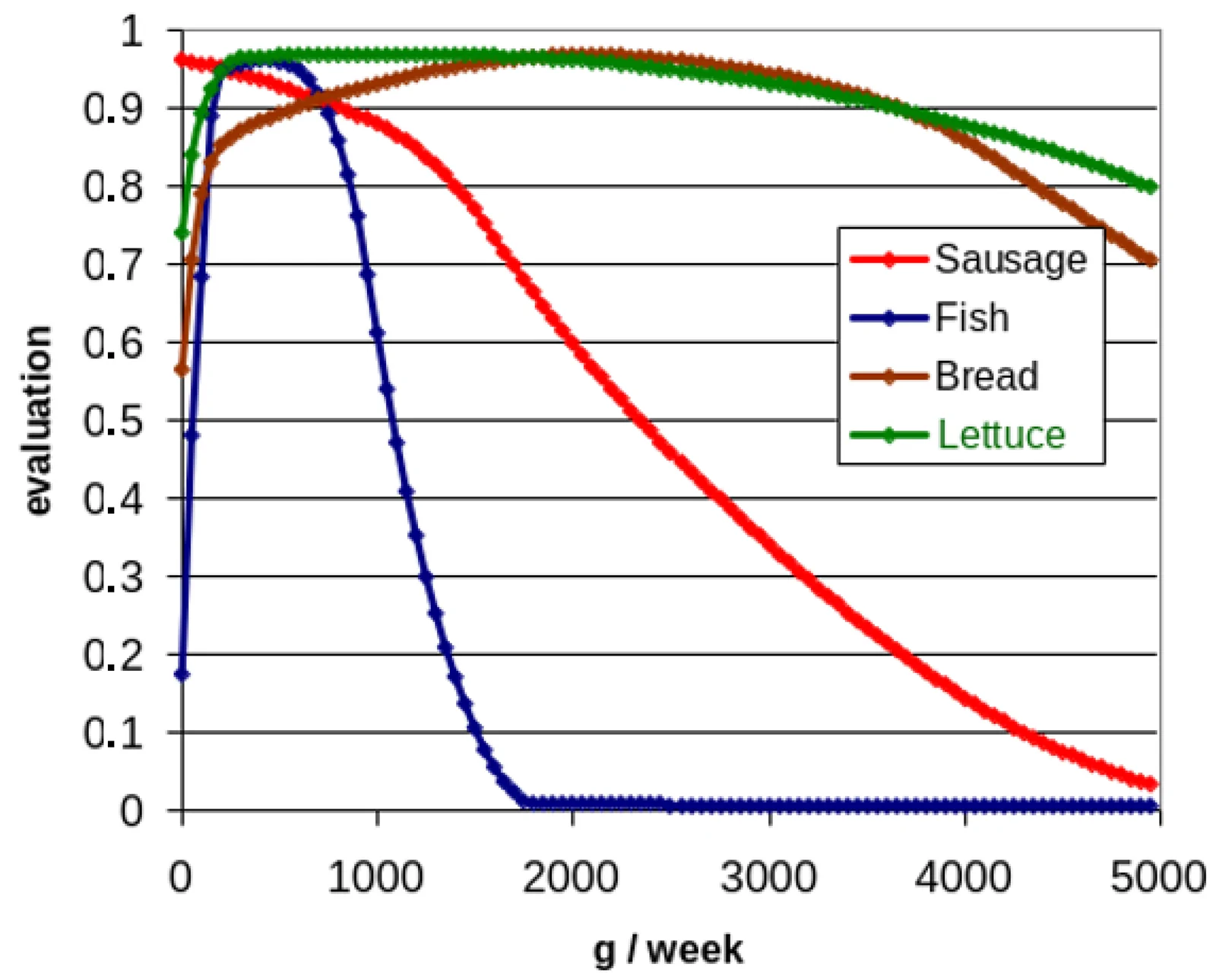
Influence of selected foodstuffs on the aggregated result.

**Figure 4:**
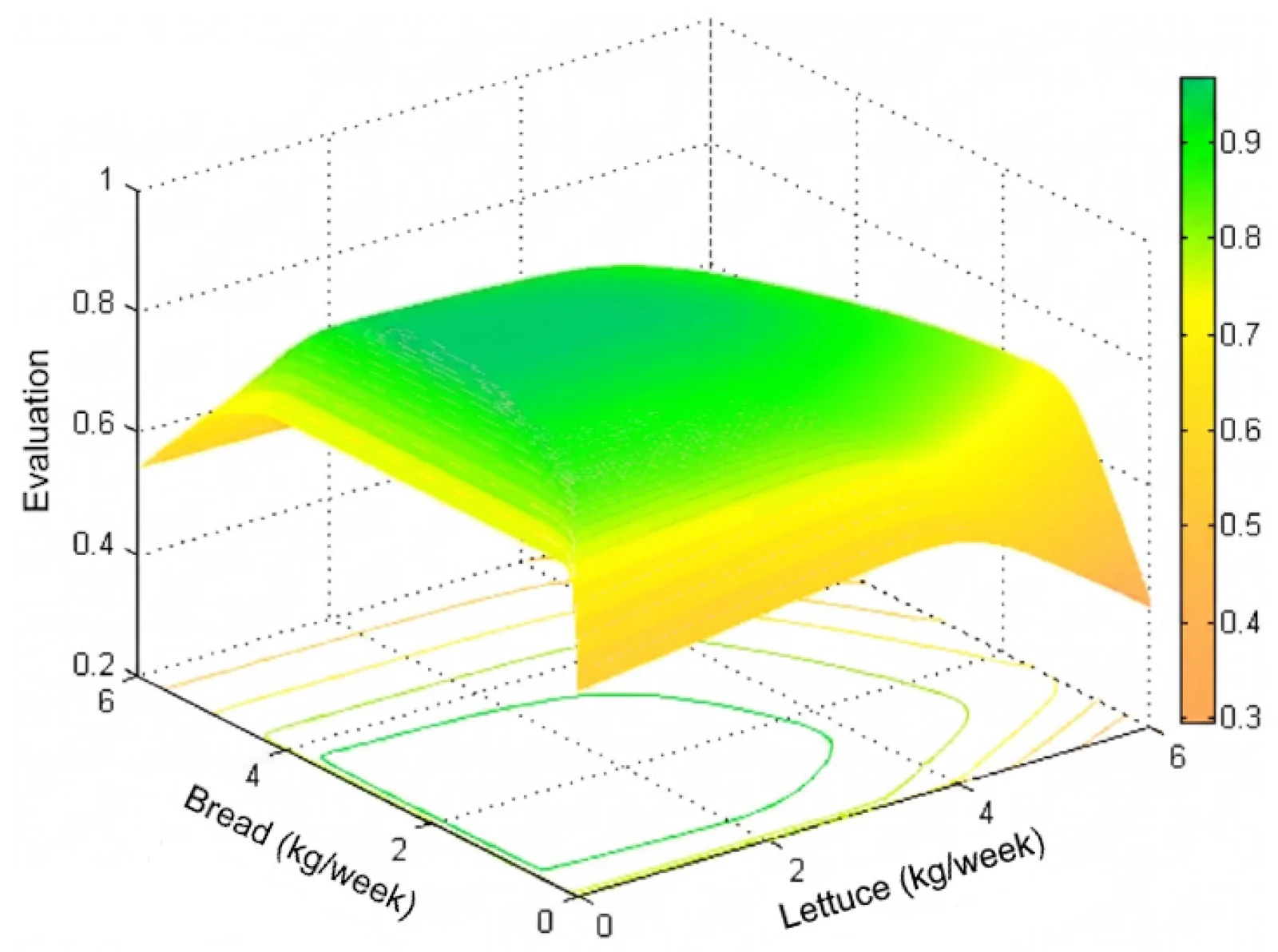
Synergistic interaction between bread and lettuce, illustrating the plateau structure of nutrition.

**Figure 5:**
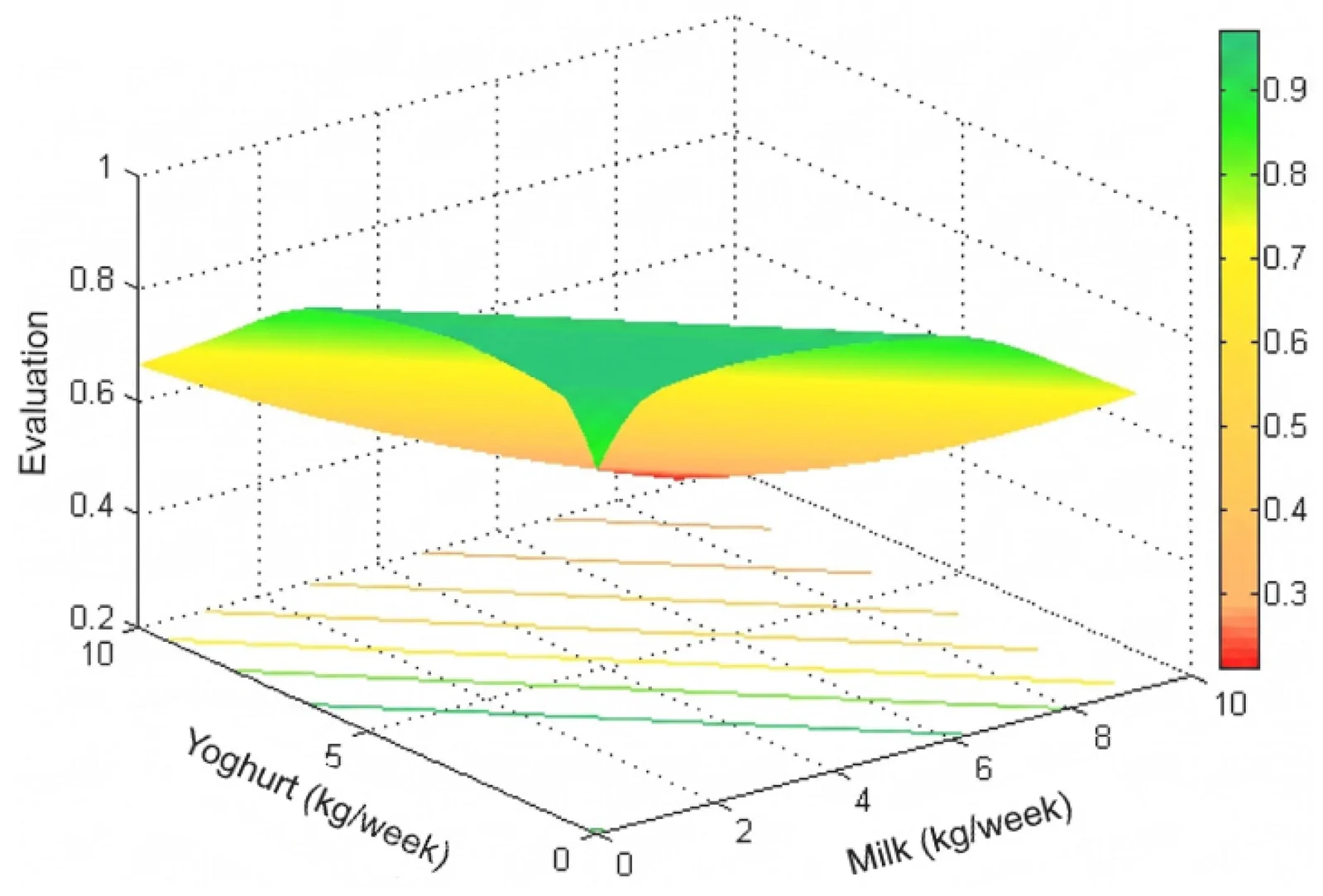
Substitution effect between two foodstuffs with similar nutrient composition.

**Table 1:** Foodstuffs retained after iterative dimensionality reduction, listed by energy contribution. BLS 3.0 codes, daily intake (g), and energy contribution (kcal/day) are shown for a reference diet of a 40-year-old man (1.72 m, 68 kg; target: 2280 kcal/day).

BLS-Code	Foodstuff	Intake/d (g)	Energy (kcal)/d
B LS B271000	Bread	468.57	661.24
B LS C350022	Rice, cooked	279.64	595.40
B LS M111000	Milk	411.43	274.17
B LS K100022	Potatoes, cooked	214.29	155.59
B LS H100000	Nuts	22.86	136.87
B LS T102022	Herring, cooked	51.43	132.79
B LS G611022	Rutabaga, cooked	210.00	74.73
B LS G560100	Tomatoes	195.71	39.27
B LS G102000	Lettuce	172.14	31.66
B LS M140300	Yogurt	42.86	29.59
B LS E100000	Eggs	8.57	11.71
B LS G490000	Garlic	0.57	0.83
B LS N110000	Water	1714.29	0.00

**Table 2:** Nutrient intake and fuzzy evaluation at the dietary optimum (13-foodstuff reduced model; reference man: 40 years, 1.72 m, 68 kg). All individual evaluations exceed 0.9; no intake exceeded an established upper limit. Aggregate Schweizer (3) score (*p* = 6): 0.941. DGE recommendation types: RI = Recommended Intake; EV = Estimated Value; GL = Guideline Value.

Nutrient	DGE-Recommendation	Intake/d	Evaluation
Energy	2280 kcal	2140 kcal	0.98
Fat, E%	<30%	18%	0.96
Carbohydrates, E%	>50%	63%	1.00
Protein/kg body weight	0.8 g/kg	1.15 g/kg	0.99
Water	2.6 L	3.28 L	1.00
Retinol equivalent	1 mg	0.925 mg	1.00
Vitamin D (Calciferol)	20 µg	13.6 µg	1.00
Vitamin E (Tocopherol)	14 mg	9.26 mg	0.95
Vitamin B1 (Thiamin)	1.2 mg	1.4 mg	1.00
Vitamin B2 (Riboflavin)	1.4 mg	1.53 mg	1.00
Niacin equivalent	15 mg	31.4 mg	0.95
Pantothenic acid	5 mg	6.73 mg	1.00
Vitamin B6 (Pyridoxin)	1.6 mg	1.95 mg	1.00
Biotin	40 µg	44.1 µg	1.00
Folic acid (total)	0.3 mg	0.49 mg	0.99
Vitamin B12 (Cobalamin)	4 µg	6.6 µg	1.00
Vitamin C	0.11 g	0.144 g	1.00
Sodium	2 g	2.08 g	1.00
Potassium	4 g	3.77 g	0.98
Calcium	1 g	1.02 g	1.00
Magnesium	0.35 g	0.378 g	1.00
Phosphorus	0.55 g	1.46 g	0.99
Iron	11 mg	12.2 mg	1.00
Zinc	14 mg	10.5 mg	0.98
Copper	1–1.5 mg	1.69 mg	1.00
Manganese	2–5 mg	6.05 mg	0.99
Fluoride	3.5 mg	1.24 mg	0.97
Iodine	0.2 mg	0.124 mg	0.95
Dietary fiber	30 g	37.8 g	1.00
Sucrose, E%	<10%	1.4%	1.00
Cholesterol	<0.3 g	0.126 g	0.99
Alcohol	<20 g	0.0 g	1.00
Saturated fatty acids, E%	<10%	6.9%	0.96
*n*-3 Fatty acids, E%	0.5%	1.2%	0.99
*n*-6 Fatty acids, E%	2%	2.2%	1.00

## Data Availability

The following data are available upon request from the corresponding author: (1) the results of the evaluation of the dietary records, including the comparison of the MIN×H and Schweizer (3) operators for *p* = 4, 6, and 8 (Excel file); (2) the full nutrient intake and fuzzy evaluation underlying Table 1 and Table 2. The original historical dietary records were stored only temporarily within the OptiDiet software during processing and optimization and were not retained in retrievable digital form thereafter.
